# Adjacent sequences disclose potential for intra-genomic dispersal of satellite DNA repeats and suggest a complex network with transposable elements

**DOI:** 10.1186/s12864-016-3347-1

**Published:** 2016-12-06

**Authors:** Eva Satović, Tanja Vojvoda Zeljko, Andrea Luchetti, Barbara Mantovani, Miroslav Plohl

**Affiliations:** 1 Division of Molecular Biology, Ruđer Bošković Institute, Zagreb, Croatia; 2 Dipartimento di Scienze Biologiche, Geologiche e Ambientali-Università di Bologna, Bologna, Italy

**Keywords:** Satellite DNA, Junction regions, Mobile elements, Sequence rearrangements, Genome evolution

## Abstract

**Background:**

Satellite DNA (satDNA) sequences are typically arranged as arrays of tandemly repeated monomers. Due to the similarity among monomers, their organizational pattern and abundance, satDNAs are hardly accessible to structural and functional studies and still represent the most obscure genome component. Although many satDNA arrays of diverse length and even single monomers exist in the genome, surprisingly little is known about transition from satDNAs to other sequences. Studying satDNA monomers at junctions and identifying DNA sequences adjacent to them can help to understand the processes that (re)distribute satDNAs and significance that evolution of these sequence elements might have in creating the genomic landscape.

**Results:**

We explored sets of randomly selected satDNA-harboring genomic fragments in four mollusc species to examine satDNA transition sites, and the nature of adjacent sequences. All examined junctions are characterized by abrupt transitions from satDNAs to other sequences. Among them, junctions of only one examined satDNA mapped non-randomly (within the palindrome), indicating that well-defined sequence feature is not a necessary prerequisite in the junction formation. In the studied sample, satDNA flanking sequences can be roughly classified into two groups. The first group is composed of anonymous DNA sequences which occasionally include short segments of transposable elements (TEs) as well as segments of other satDNA sequences. In the second group, satDNA repeats and the array flanking sequences are identified as parts of TEs of the *Helitron* superfamily. There, some array flanking regions hold fragmented satDNA monomers alternating with anonymous sequences of comparable length as missing monomer parts, suggesting a process of sequence reorganization by a mechanism able to excise short monomer parts and replace them with unrelated sequences.

**Conclusions:**

The observed architecture of satDNA transition sites can be explained as a result of insertion and/or recombination events involving short arrays of satDNA monomers and TEs, in combination with hypothetical transposition-related ability of satDNA monomers to be shuffled independently in the genome. We conclude that satDNAs and TEs can form a complex network of sequences which essentially share the propagation mechanisms and in synergy shape the genome.

**Electronic supplementary material:**

The online version of this article (doi:10.1186/s12864-016-3347-1) contains supplementary material, which is available to authorized users.

## Background

Satellite DNAs (satDNAs) and transposable elements (TEs) are the two most abundant classes of repetitive sequences in eukaryotic genomes [1]. SatDNAs are defined as tandemly repeated, non-coding DNA sequences that form megabase-long arrays located in heterochromatin. A peculiarity of satDNAs is the concerted evolution of repeat units accompanied by extremely dynamic array expansions and contractions, a process driven by diverse mechanisms of non-reciprocal sequence exchanges [2]. In contrast, a major feature of TEs is genome mobility, self-replication and formation of interspersed repeats [3, 4]. Diverse TEs, which move either autonomously or by using enzymes of other autonomous elements, constitute a complex network of interlinked sequences. As a result, a significant fraction of the genome could be derived from TEs and sequences that resulted from their deterioration (for example, [5–7]).

Despite conceptual differences, there are many reports showing that satDNAs and TEs can share a close sequence relationship, although involving different DNA segments (reviewed in [8]). For example, TEs can contribute to formation of satDNAs by tandem amplification of a whole element or its part [9–11]. Short arrays of tandem repeats can also be found within TEs, and in some cases these repeats appear to be the building blocks of satDNAs [12–17].

In addition to localized organization into long homogeneous arrays of heterochromatin, growing evidence suggests a much broader, genome-wide distribution of satDNAs [10, 18, 19]. Analysis of sequence variability performed on the 1.688 satDNA of *Drosophila* [20] and on satDNAs of the beetle *Tribolium castaneum* [19] showed that satDNA copies located in euchromatic chromosomal domains evolve under similar rules as their counterparts in heterochromatic compartments. A heat-stress induced regulatory mechanism of transient genome-wide heterochromatinization has been recently proposed in *T. castaneum* as a possible functional role of isolated euchromatic satDNA copies [21].

A direct consequence of extensive shuffling of satDNA repeats are numerous junctions with other sequences that can track processes forming the current pattern. Although a large number of satDNAs have been described in detail, there is still only limited information about the molecular characteristics of such junction sites. SatDNA monomers at array ends often show enhanced level of decay, a phenomenon considered to be a consequence of the lack of sequence homogenization of terminal repeats by unequal crossover [22–25]. However, observed exceptions to this rule can be either because junctions were recently formed or because monomers are not homogenized by unequal crossover at all, for example, if arrays are too short and/or isolated from the rest [19, 26, 27], or are supposed to evolve under constraints [20, 21]. In any case, considering the available data, satDNA sequence ends mostly form well-defined junctions, irrespective of whether they are between different satDNAs [26–29], satDNAs and TEs [22, 30], or satDNAs and other sequences [20, 31].

Bivalve molluscs represent a large class of marine and freshwater invertebrates with more than 8000 extant species. Despite numerous specificities and importance in ecology, aquaculture and fisheries, this group of organisms is relatively poorly explored at the genome level. Whole genome sequences are available only for the Pacific oyster *Crassostrea gigas* [32] and the pearl oyster *Pinctada fucata* [33]. A dozen satDNAs have been characterized in bivalves, and some of them were found to be widespread in a number of species and persistent over long evolutionary time [34, 35]. In contrast to satDNAs, only a few non-autonomous miniature inverted-repeat transposable elements (MITEs) have been described and classified in molluscs, such as modular elements of the *pearl* family, characterized by internal repeats similar to some satDNA monomers [12, 34, 36]. In a *pearl*-related element of the clam *Donax trunculus* we dissected the modular composition and possible mechanisms that drive rearrangements of internally located tandem repeats [14].

Here, we study randomly selected satDNA-harboring genomic fragments in 4 mollusc species. Two of the species are related and belong to the same superfamily (Veneroidea): the Manila clam *Ruditapes philippinarum*, a cosmopolitan invasive species, and its retreating counterpart, the grooved carpet shell *R. decussatus*. The other two are distantly related: the clam *D. trunculus* (superfamily Tellinoidea) and the oyster *Crassostrea gigas* (superfamily Ostreoidea). The abundant satDNAs already characterized in the selected mollusc species, the peculiar long-term conservation of some satDNAs and their association with MITEs make an interesting framework for studying interrelations between satDNAs and other genomic sequences. Our analysis has focused on characterization of peripheral and/or interspersed satDNA monomers (i.e., located at the end or outside of typical arrays of tandem repeats) and on annotation of flanking DNA sequences. The aim of this study is to explore the molecular traits of junctions, and how such satDNA repeats may participate in shaping the genome as a whole. To the best of our knowledge, this is the first report characterizing transitions in a random set of hybrid genomic fragments containing both satDNAs and non-satellite genomic sequences.

## Methods

### Construction of partial genomic libraries and colony lift

Genomic DNA from the examined mollusc species was isolated according to the standard phenol/chloroform protocol, slightly modified for DNA isolation from adult specimens of *D. trunculus* [37]. Following the strategy described by Biscotti et al. [38], genomic DNA of *R. decussatus* and *R. philippinarum* was partially digested (10 μg of DNA, 37 °C/5 min) with 5 U of *Alu*I restriction endonuclease or, in the case of *D. trunculus* genomic DNA, with 5 U of *Alu*I or *Bam*HI restriction endonucleases (Fermentas). The obtained fragments were ligated into the pUC19 vector, and transformed into *E. coli* DH5α competent cells (Invitrogen) following which cells were grown on ampicillin-selective plates. After colony transfer, positively charged membranes (Amersham) were probed with *Alu*I-digested digoxigenin-labeled genomic DNA of the corresponding species. Hybridization with complete genomic DNA develops a more intensive hybridization signal in sequences present in large copy number compared to those present in a single copy. Colony hybridization was conducted under 65 °C in 20 mM sodium phosphate buffer (pH 7.2), 20% SDS, allowing ~80% sequence similarity. Stringency washing was performed in 20 mM sodium phosphate buffer, 1% SDS, at a temperature three degrees lower than the hybridization temperature. To detect the hybridization signal, membranes were incubated with anti-digoxigenin alkaline phosphatase conjugate and chemiluminescent signals induced by CDP-Star (Roche) were captured on X-ray films (Amersham).

### Sequencing and sequence analysis

Plasmid DNA with selected inserts was isolated from *E. coli* clones and sequenced at Macrogen Inc. In the case of Dt-BIV160, additional repeats were amplified from genomic DNA by PCR, using the primer pair, bivF: TACATAGACTTATATAGGGAAAATC, and bivR: TTTGACCCCAGGGGAATAATT. PCR amplification was performed with initial denaturation at 94 °C for 5 min, 30 cycles of 94 °C for 30 s, 55 °C for 30 s, 72 °C for 30 s, and final extension at 72 °C for 7 min. All PCR products were cloned into the pGEM-T Easy vector system (Promega), and multimer-containing clones sequenced.

Sequences submitted to GenBank obtained the following accession numbers KU682284 - KU682293, KU682294 - KU682299, KU682300 - KU682313, KU682314 - KU682355. Clones DTC17AluF, 84–35, and DTC52Alu already exist in the database with the corresponding accession numbers: KC981731, KC981682, and KC981735 (Additional file 1: Table S1). Sequences of fragments shorter than 200 bp could not be deposited in GenBank but can be obtained upon request.

Sequence editing, alignments and local BLAST searches were performed using the Geneious 5.4.3 program (Biomatters Ltd.). Tandem repeats were defined using TRF [39] default parameters and manually adjusted where needed. SatDNA consensus sequences were built according to the majority principle, by combining entries deposited in databases and monomers sequenced in this work. Substructures, repeats and motifs were searched with appropriate applications within the Oligonucleotids repeats finder online tool (http://wwwmgs.bionet.nsc.ru/mgs/programs/oligorep/InpForm.htm). The CENSOR online tool [40] was used for screening query sequences against a repetitive DNA collection deposited in Repbase.

### Search for Cg170 satDNA in the C. gigas genome assembly

The *C. gigas* genome assembly (oyster.v9; [32] was analyzed using Geneious v6.1.8 (Biomatters Ltd.). The Cg170 monomer sequence deposited in the Repbase under entry “SATREP” was used as a query in BLAST screening. From the obtained results, the first 10 scaffolds revealing hits with the best E-values (4.2e-86) were chosen for a more detailed analysis (the top 10 set). Furthermore, additional 10 sequences were selected among all obtained hits using a local script which shuffles sequences in a random way (the random 10 set). The positions of retrieved *C. gigas* genomic fragments on scaffolds is shown in Additional file 2: Table S2. Up to 500 bp long sequences that flank satDNA repeats from both sides were included in all analyses (left; LF500, and right; RF500). The extracted LF500 and RF500 sequences were compared among themselves and with the Repbase using CENSOR [40].

### Phylogenetic analyses

SatDNA monomers have been aligned using the Muscle algorithm implemented in Mega v.6 [41]. The best substitution model (T92 + G for all datasets) and Maximum Likelihood trees have been calculated with Mega v. 6; nodal support has been obtained with the bootstrap method after 100 replicates. For comparative purposes, the following sequences drawn from the Genbank were included: the *phBgl*II400 satDNA dimer (acc. no. U80936; [42]), DTHS3 monomers (acc. nos. X94611, X94540, X94542; [43]), and monomers from *R. philippinarum* and *R. decussatus* BIV160, *D. trunculus* pDTE, and oyster *Hind*III satDNAs, already included in the previous satDNA analysis [34].

### Breakpoint annotation and distribution analysis

Breakpoint nucleotides have been annotated by aligning segments composed of satDNA monomers and their adjacent anonymous sequences identified in the initial BLAST searches with consensus sequences of corresponding satDNA families arranged in artificial dimers and/or trimers. When necessary, alignments were adjusted manually. The sudden drop in sequence similarity could be identified visually, and was verified by determining nucleotide position in which similarity drops below 60% (in a minimal stretch of 10 nucleotides).

In order to test if observed breakpoints are distributed randomly across the sequence or are preferentially clustered in restricted regions, we simulated a null breakpoints distribution to compare with the observed data. For each satDNA family, we drew the same number of breakpoints as in the observed data but occurring randomly along the sequence (as it would result from a discrete uniform distribution); this was done for 100,000 replicates. Then, the average pairwise distance (calculated in number of bp) between breakpoints was calculated for each replicate and used to build a null distribution which approximates a normal distribution. Finally, we checked if the average pairwise distance between observed breakpoints falls within or outside the distribution of the simulated data. Statistical significance was assessed by a one-sample Z-test. If the average pairwise distance between observed breakpoints is significantly lower than the average pairwise distance between simulated breakpoints then the observed breakpoints are considered to cluster in a restricted region of the satDNA monomer.

## Results

### Collection of repetitive DNA-containing genomic fragments

DNA fragments enriched in repetitive sequences were detected in partial genomic libraries of *R. philippinarum*, *R. decussatus* and *D. trunculus* in the course of several rounds of cloning and hybridization with total genomic DNA as a probe. Using this approach we collected between 50 and 70 clones of potential interest for each species. After sequencing, we focused on 36 fragments, selected on the basis of similarities with already published satDNAs. A search for tandem repeats that would indicate the presence of yet uncharacterized satDNAs gave no results in this set.

Cloned fragments were up to 4 kb long (1 kb on average), 19 are made exclusively of satDNAs, while 17 are hybrids of satDNA, TE(s) and anonymous sequences (Additional file 1: Table S1). TE-related sequences mostly appear as short segments (44–192 bp) of moderate similarity (between 66% and 84%) to DNA transposons and/or LTR-retrotransposons. An exception is the recently characterized short interspersed element (SINE) named RUDI [44], present as a complete copy in D12. In addition, a search for uncharacterized repeated elements by comparing all sequenced fragments revealed two putative MITEs in *D. trunculus*, DTCM1 and DTCM2, preliminarily classified based on terminal inverted repeats and target site duplication (TSD).

### phBglII*400 satDNA*

**Fig. 1:**
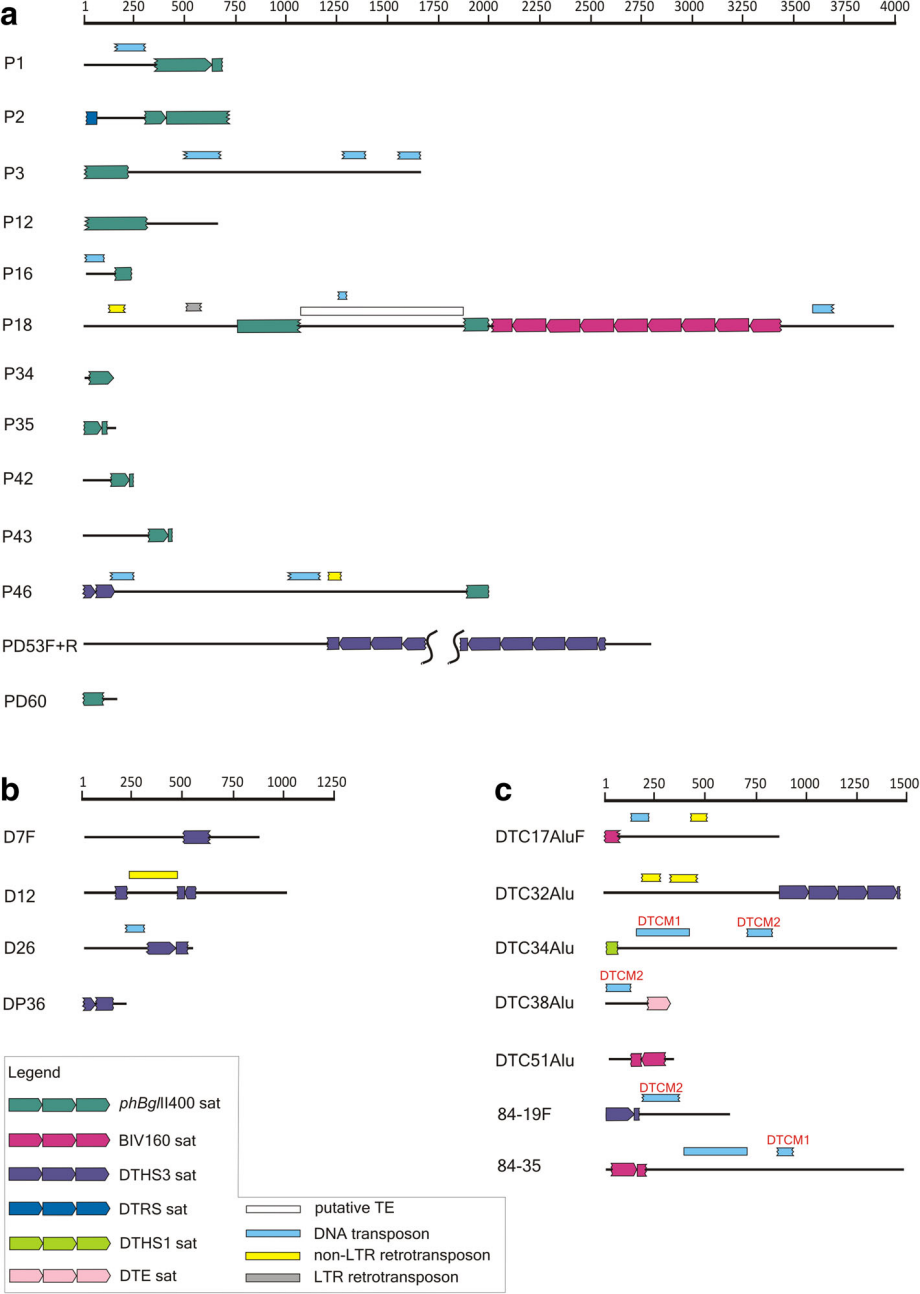
Schematic presentation of genomic fragments cloned from (**a**) *R. philippinarum*, (**b**) *R. decussatus*, and (**c**) *D. trunculus*. Black lines represent cloned genomic fragments with satDNAs indicated as arrowed rectangles. Arrow position indicates the monomer frame according to original papers [34, 37, 43, 45–48]. Colored rectangles above the line represent regions of similarity with TEs revealed in the database search. Only segments with highest sequence similarity are shown (the complete list is shown in Additional file 1: Table S1a-c). Putative novel elements DTCM1 and DTCM2 are named above rectangles. Fragment PD53 is not overlapped because of difficulties in the assembly of DTHS3 satDNA array. Waved rectangle ends indicate truncated sequences. Scale indicates fragment length in base pairs

From *R. philippinarum* we isolated 12 genomic fragments that harbor *phBgl*II400 satDNA [42] associated with other sequences (Fig. 1a, and Additional file 1: Table S1a). Despite the relatively short length, some cloned fragments include more than one satDNA. P2 holds one complete *phBgl*II400 monomer and a short 43 bp long segment 86% similar to the DTRS satDNA, detected previously in *D. trunculus* [45]. In addition to *phBgl*II400 satDNA and parts of different TEs, a 2 kb long fragment P46 harbors the DTHS3 satDNA ([43]; see also below). The most complex sequence in this set is in the clone P18. It contains one copy of a *PhBgl*II400 monomer precisely split by an 806 bp long sequence flanked by TGATC direct repeats. As this pentanucleotide is part of the *phBgl*II400 monomer, it could be proposed that direct repeats are TSDs formed upon insertion of the interrupting sequence. In addition, P18 contains a 1426 bp long array made up by 9 consecutive monomers of BIV160 satDNA [34], separated only by a short segment (22 bp) from the split *phBgl*II400 monomer (see below for the description of BIV160).

**Fig. 2:**
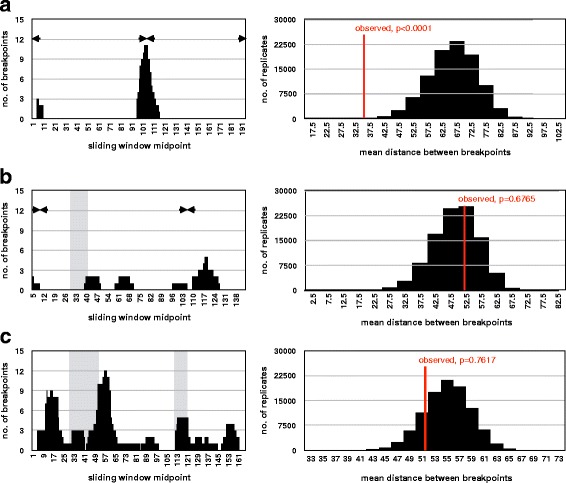
Positioning of satDNA junction nucleotides (*left*) and clustering statistical analysis (right) in (**a**) *phBgl*II400 satDNA, (**b**) DTHS3 satDNA, (**c**) BIV160 / Cg170 satDNAs. Light grey shaded areas in left panels indicate conserved blocks, while arrowheads mark the palindromes. In the right panel, histograms represent the distribution of simulated average distance between breakpoints, while the thin line represents the value of the observed distribution (with associated *p*-value)

Phylogenetic analysis did not reveal any specificity of *phBgl*II400 monomers reported in this work, and only confirmed the already reported partition into two subfamilies (Additional file 3: Figure S1a). The junction ends of *phBgl*II400 monomers are abrupt, as evidenced by a sudden decrease in similarity (from >90% to 45–50%) observed in comparisons with the *phBgl*II400 satDNA consensus sequence. Because of abrupt ends, we were further interested to see if transition sites are clustered and if they could be associated with any specific sequence feature of the satDNA monomer. In this analysis we used two aligned halves of *phBgl*II400 satDNA monomer consensus sequence, due to internally repetitive character of the monomer [42]. Mapping the breakpoint nucleotides clearly revealed their evidently non-random positioning (Fig. 2a, and Additional file 4: Figure S2a). The majority of breakpoints (13/16) are associated with the longer palindrome (12 bp), while the rest map within the shorter (10 bp) palindrome sequence.

### DTHS3 satDNA

Another detected satDNA is DTHS3 [43], recovered in genomic fragments from all three studied species, and found associated with non-DTHS3 sequences in eight clones (Fig. 1, and Additional file 1: Table S1). As described for the *phBgl*II400 satDNA, DTHS3 is also linked with fragments of diverse TEs, other satDNAs and anonymous sequences. Specifically, DTHS3 forms an array of four tandem repeated monomers in the fragment DTC32Alu of *D. trunculus*, while short segments in the array flanking region can be assigned to retrotransposons (Fig. 1c). Fragment D12 (Fig. 1b) harbors one complete DTHS3 monomer which has been split by the insertion of a full-length copy of the SINE element RUDI [44]. Although it was previously reported that RUDI regularly forms TSD sequences upon insertion, they were not found in this case.

Sequence similarity among DTHS3 monomers obtained in this work and those cloned earlier from a restriction-digestion band of *D. trunculus* [43] varies between 67.6%–95.2%. Phylogenetic analysis clearly resolved groups according to the species of origin, except for one *R. philippinarum* monomer (Additional file 3: Figure S1b). Alignment of species-specific consensus sequences revealed a 15 bp long segment with lower variability compared to the rest of the monomer sequence, as well as two short palindromes, six and eight nucleotides long (Additional file 4: Figure S2b). As for *phBgl*II400 satDNA, the junctions between DTHS3 satDNA and other sequences revealed abrupt transitions. However, in this case, satDNA junction positions are scattered randomly along the monomer sequence (Fig. 2b, and Additional file 4: Figure S2b).

### BIV160 satDNA

Among the studied genomic fragments obtained from *R. philippinarum* and *D. trunculus* we identified copies of BIV160 satDNA in four clones (Fig. 1a, c, and Additional file 1: Table S1a, c). Moreover, sequencing from colony hybridization experiments on *D. trunculus* resulted in 3 additional clones containing only BIV160 satDNA (DTC4Alu, DTC50Alu, and DTC52Alu; Additional file 1: Table S1c). Due to the fact that BIV160 remained undetected in *D. trunculus* during initial study of this satDNA [34], specific primers were constructed to obtain additional monomer variants. In total, 42 monomers were isolated by PCR amplification of *D. trunculus* genomic DNA and included in the subsequent analyses.

In the phylogenetic analysis, *R. philippinarum* BIV160 monomers intermingle with monomers characterized previously, without indicating any specific clustering [34] (Additional file 3: Figure S1c). However, *D. trunculus* monomers (regardless of how they were obtained) group separately from other BIV160 sequences. They also remained separated from the related pDTE satDNA, characterized earlier in the same species [37], thus representing a distinct, species-specific clade. The consensus sequence derived from monomers recovered from *D. trunculus* in this work is 85% similar to the BIV160 consensus sequence determined earlier [34]. Nonetheless, the two conserved sequence segments are retained in all variants (Additional file 4: Figure S2c).

Among hybrid genomic fragments containing BIV160 satDNA and other sequences, the ~4 kb-long *R. philippinarum* fragment P18 incorporates an array of nine BIV160 repeats adjacent to the *phBgl*II400 satDNA monomer described above (Fig. 1a). A *D. trunculus* composite fragment 84–35 contains one complete BIV160 monomer which with a couple of nucleotides extends into the subsequent monomer, a full-length copy of DTC84 (a MITE element related to the *pearl* family; [14]), and one truncated copy of a putative MITE element DTCM1 (Fig. 1c). The BIV160 monomer and the two MITEs are separated from each other by short segments of anonymous sequences. Junction sites of BIV160 monomers are commented below, together with those of Cg170 satDNA.

### Cg170 satDNA

**Fig. 3:**
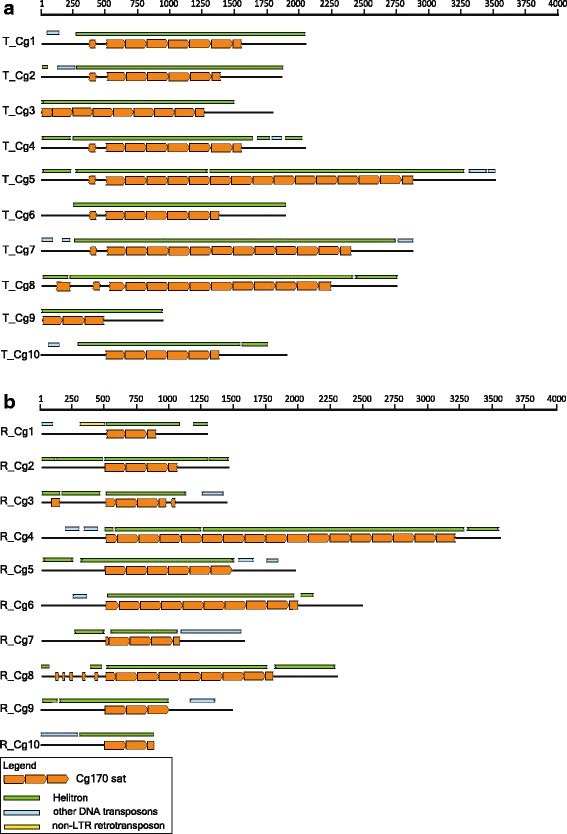
Schematic presentation of (**a**) top 10 (T_Cg1-10) and (**b**) random 10 (R_Cg1-10) fragments selected from *C. gigas* genome assembly. Tandemly repeated Cg170 monomers (*orange arrows*) are flanked with up to 500 bp long sequences (*black lines*). The monomer sequence frame is presented according to the BIV160 consensus [34], arrowheads showing orientation. Colored rectangles above genomic fragments represent regions of similarity with TEs (for details see Additional file 1: Table S1d). Waved rectangle ends indicate truncated sequences. Scale indicates fragment length in base pairs

Cg170 satDNA of *C. gigas* [46, 47] is related to the BIV160 satDNA (64.6% similarity), and they both share two conserved sequence motifs ([34] (Additional file 4: Figure S2c). Based on this, we decided to examine a sample of Cg170 repeats and their flanking sequences mined from the assembled *C. gigas* genome [32]. Two sets of Cg170-containing fragments were analyzed; 10 were selected according to the best E-value, and another 10 were selected randomly among positives obtained in the search (Fig. 3, and Additional file 1: Table S1d). No substantial differences could be observed in the architecture of the “top 10” (marked T_Cg1-10) and “random 10” (marked R_Cg1-10) fragments. Arrays of Cg170 satDNA obtained in this search contain up to 17 tandem repeated monomers. In the phylogenetic tree, oyster Cg170 monomers and related oyster *Hin*dIII satDNA [48] cluster separately from BIV160 satDNA [34]. Moreover, although Cg170 sequences showed limited intermingling with *Hin*dIII monomers, top 10 and random 10 datasets cannot be resolved (Additional file 3: Figure S1c).

According to overall similarity comparisons, analyzed Cg170 arrays as well as most of their flanking sequences can be mainly assigned to DNA transposons of the Helitron superfamily (87–94% similarity; detailed characterization of these elements in *C. gigas* genome will be presented elsewhere). Because of shared junction nucleotides in satDNA monomers and because of high sequence similarity in flanking sequences, most of the fragments studied in this set likely emerged in amplification of one master element, differing only in the copy number of Cg170 monomers in the internal array (Fig. 3, and Additional file 4: Figure S2c). All junction ends detected on the same nucleotide we therefore treated in further analyses as a consequence of a single mutational event.

Apart from Helitrons, some Cg170 flanking sequences share similarity with short segments of other mobile elements interspersed among anonymous sequences (Fig. 3), thus suggesting a pattern similar to that described in the sections above. One, probably recent, recombination event can be inferred in the left flanking of R_Cg10, where about 300 bp long segment sharing 94% similarity to Helitron-N2_CGi is followed by a 170 bp long stretch 96% similar to the Kolobok-N2_CGi transposon (Fig. 3, and Additional file 1: Table S1d).

**Fig. 4:**
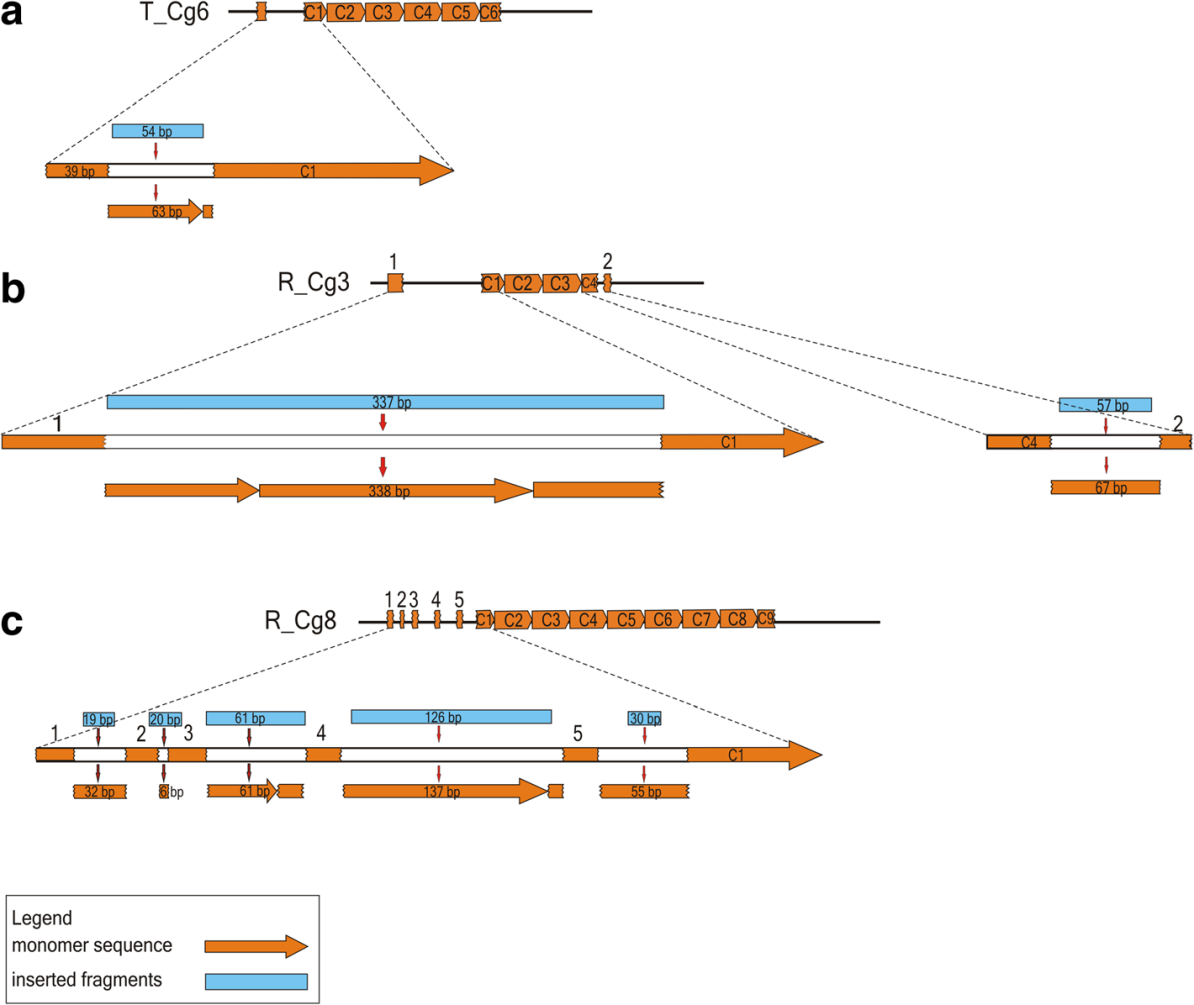
Detailed schematic presentation of interrupted monomers positioned in flanking regions of some Cg170-containing fragments; (**a**) in T_Cg6; identical junction positions of monomer segments are also in fragments T_Cg1, T_Cg2, T_Cg4, T_Cg5, T_Cg7, and T_Cg8, (**b**) in R_Cg3 and (**c**) in R_Cg8. In the first line of each drawing is the complete fragment with indicated satDNA monomers (yellow rectangles). In the second line is enlarged diagram of interrupted satDNA monomers with gaps indicating locations of extruded monomer segments, shown below. Blue rectangles represent sequence segments that are actually replacing corresponding parts of the monomer. All rectangle and arrow lengths are presented in scale

In addition to TEs and anonymous sequences, detailed insight into Cg170 flanking regions also revealed remnants of satDNA monomers. In the T_Cg1-10 set, a 39 bp long monomer segment (>80% similarity with the Cg170 consensus) is positioned in the left flanking sequence in seven fragments (Fig. 3a). It is separated from the first monomer in the Cg170 array by a 54 bp long spacer (Fig. 4a). Interestingly, a missing monomer segment that would arrange a continuous satDNA sequence is of similar length, 63 bp, suggesting a replacement event in which part of the monomer was replaced by a segment of another sequence. Segment position and ends are the same in all studied fragments, indicating again that the described arrangement is a consequence of a single event. One additional monomer segment, arranged in a similar way, is observed in the fragment T_Cg8 (Fig. 3a).

Genomic fragments R_Cg3 and R_Cg8 also harbor monomer segments located in array flanking sequences (Fig. 3b). These segments are between 19 and 64 bp long, and show about 90% similarity to the Cg170 consensus sequence. Their distribution in flanking regions identified them as remnants of whole-length monomers in continuity with repeats in the array but disrupted by unrelated sequences (Fig. 4b, c). As in the above example, the missing parts of satDNA monomers are similar in length to the inserted satDNA-unrelated sequence, indicating cut and replace events.

Because BIV160 and Cg170 satDNAs are related and can be considered members of the same family, the breakpoint analysis has been carried out for both of them together. In all examined repeats junctions are mostly positioned in close proximity to or within the conserved sequence blocks, characteristic for this family of satDNA repeats. Interestingly, although some clustering in these regions could be inferred, statistical analysis could not support significance of this distribution (Fig. 2c, and Additional file 4: Figure S2c).

## Discussion

A broadly accepted observation is that monomers of different satDNAs have very little or nothing in common, although they are often characterized by distinct sequence features, such as conserved motifs, inverted repeats, and palindromes (reviewed in [49]). A possible role of these structural elements could be in providing signals that promote mechanisms involved in the process of rapid propagation of satDNA repeats, either within arrays or across the genome. Illegitimate recombination and transposition-related mechanisms are considered as the most representative of this process. For example, shared sequence motifs were identified as triggers of recombination between arrays of human alpha satDNA [50, 51].

Studies of the transition between satDNAs and other genomic sequences enable characterization of satDNA sequence ends and associated features within the sequence. Although accumulation of sequence divergences in satDNA monomers at array ends can lead to loss of monomer identity and consequently can blur the transition site [22–25], there are also examples of abrupt switches from a satDNA to another sequence (for example, [20, 26, 27]). Phylogenetic analysis shows that bordering monomers examined in this work do not bear any notable differences with respect to the pool of variants, allowing assumed reliable assignment of satDNA sequence ends. In this regard it can be assumed that presented cloned fragments do not contain borders of long satDNA arrays that would be maintained by unequal crossover [22–25] but are rather representatives of monomers or their short arrays positioned on diverse genomic locations outside of the main chromosome cluster.

Although the number of studied satDNA junctions is still rather low, the available information suggests that satDNAs do not share any common architecture at the point of transition. In the parasite wasp for example, palindromes were detected at junctions between two satDNA arrays, while short stretches of sequence similarity link satDNA with a TE [22, 28]. In *Drosophila*, it has been proposed that illegitimate recombination mediated by local tracts of sequence similarity can lead to satDNA insertions into a novel genome environment, resulting in abrupt switches and interspersion of arrays [27]. Although no sequence feature could be identified in junctions between satDNAs of the beetle [26], recombination sites were localized within a 20–30 bp long segment, a stretch of comparable length as those observed in human alpha and *Drosophila* satDNAs [27, 50].

In line with previous examples, each satDNA in the studied mollusc species shows its own pattern of transition sites (the list of satDNAs retrieved in this work is shown in Additional file 5: Table S3). Junctions are (i) clearly associated with the palindrome in phBglII400 monomer, (ii) unrelated with any observed sequence feature and distributed along the whole monomer sequence in DTHS3 or (iii) are loosely grouped mostly around the longer of the two conserved sequence motifs in the related pair of BIV160 and Cg170 satDNAs. Furthermore, there is no indication of species-specificity when the same satDNA is shared among examined organisms.

Information about junction sites can also be obtained by studying short satDNA-like arrays (up to six monomers long), which are part of the modular MITE elements of the *pearl* family, widespread in bivalve molluscs [12]. In the DTC84 element of *D. trunculus*, a conserved palindrome is located near the repeat junction in all examined element copies [14]. Repeats of the element CvA of *C. virginica* are conserved in an equivalent location, but instead of a palindrome they hold there a short microsatellite-like segment [12]. It must also be mentioned that tandem repeats of the CvA element are related to BIV160 and Cg170 satDNA monomers. BIV160 and Cg170 are parts of a large family of satDNA sequences with monomers that, despite sequence divergence, share two well-conserved sequence motifs [34]. In spite of this, our present study could not provide any statistical support in favor of a link between junction sites and conserved motifs in BIV160 and Cg170 monomers, questioning their relevance in formation of satDNA junctions. It can thus be concluded that palindromes, inverted repeats, and conserved sequence motifs can signify, but are not the necessary determinant of junctions between satDNAs and adjacent sequences.

The second question we assessed in this work concerns the composition of DNA sequences adjacent to satDNAs. One set of our results shows satDNAs linked to anonymous, probably non-repetitive sequences occasionally interrupted with short segments annotated as remnants of diverse TEs. However, in the case of *C. gigas* we found short arrays of Cg170 satDNA that, together with flanking regions, likely represent constitutive parts of Helitron TEs. In line with this observation, sequences related to a satDNA originally isolated in the pilgrim scallop *Pecten jacobaeus* were found incorporated in the *C. gigas* genome in segments similar to non-autonomous DNA transposons, suggesting a general importance of TEs in the spread of tandem repeats [35].

Species-specific differences observed here in the associations of satDNAs and other sequences can be the result of two different study approaches, *in silico* (*C. gigas*) and experimental (*R. philippinarum*, *R. decussatus* and *D. trunculus*). Among the surveyed species, only the *C. gigas* genome has been sequenced and assembled [32]. The experimental approach has evident limitations, and can be easily biased, for example, due to the construction of genomic library and the library size. On the other hand, limitations in sequence assemblies regularly make large satDNA domains excluded from datasets, and, as a consequence, short arrays such as those of Cg170 satDNA may become apparently “enriched” in the assembled genome (for the discussion about this point, see [19]). Sets explored in this survey are of comparable size and represent a kind of genomic cross-section that can be indicative of at least some patterns of associations between satDNAs and other genomic sequences.

Complexity and diversity of flanking sequences contrast with low variability of satDNA monomers located at or near junctions, which is similar to the general variability within each satDNA. This can be the result of recent waves of insertions involving satDNAs associated with TEs, and accompanied by rapid decomposition of inserted components. Although only speculative at the moment, this hypothesis can be supported by the observation that over 80% of deletions detected in the *C. gigas* sequenced genome overlap with TEs, thus indicating their high recombination potential which leads to efficient loss of element structure [32]. In TE-constitutive arrays of tandem repeats, deleterious events can result in copy number alterations and rapid shrinking until the size of a monomer or a monomer segment, as in the scenario describing dynamics of internal repeats in the *pearl*-like element DTC84 [14]. Low sequence variability of satDNA monomers has also been observed when their short segments are located in the proximity of genes. It was hypothesized that low sequence variability can in this case be a consequence of possible roles in gene regulation and/or chromatin structure [20, 21, 52].

Insertion and accumulation of mobile elements into satDNA arrays has been detected in diverse organisms [25, 53, 54]. Multiple insertions were also reported, for example a MITE and a *mariner*-like element were found integrated into satDNA of the ant, and satDNA-integrated MITE is a hot-spot for further insertions of *mariner* and other elements [30]. Some of the mollusc satDNA monomers detected in this work are clearly interrupted with inserted sequences, and here two types of events are distinctive. The first type is as expected; satDNA monomers are precisely split by putative mobile elements (RUDI and an uncharacterized segment in P18), indicating that they became inserted by a common reverse transcriptase-dependent copy-and-paste event [55], resulting in integration without any loss in the host sequence. The second type of insertion, observed in Cg170 satDNA, is based on excision of a target sequence segment and its replacement by an invading sequence of a similar length (cut-and-replace).

A cut-and-replace event in which two divergent satDNA monomers of the same length precisely replace each other was recently described in a nematode satDNA [56]. The switch occurred at a sequence motif similar to the 17 bp long CENP-B box of human alpha satDNA, common for both monomers. The CENP-B box of human alpha satDNA binds the CENP-B protein [57], broadly distributed in vertebrates and invertebrates, and related to transposases of the *pogo* family [58, 59]. It was therefore suggested that replacement of nematode satDNA monomers occurred in an event similar to transposition, although the nature of this mechanism remained unknown [56]. In the present study, no particular sequence motif could be revealed at sites of replacement of Cg170 satDNA segments. However, the pattern of disrupted monomers could be formed according to a similar scenario, in which an extraneous sequence invades Cg170 satDNA monomers resulting in excision of satDNA segments. Based on the analogy with nematode satDNA, it can be further speculated that excised satDNA segments might subsequently act as small units that can be spread throughout the genome by invading other sequences.

## Conclusions

Studying satDNA monomers associated with other genomic sequences and annotating their junctions can help to understand processes that lead to interspersion of satDNA repeats, and conceive how they participate in shaping the genome. In the studied sets of satDNA monomers, junction nucleotides revealed either localized or dispersed positioning, showing that sequence features such as palindromes or conserved sequence motifs are not indispensable elements of the transition site. Adjacent to satDNAs are found (i) anonymous sequences interspersed with short segments of diverse TEs and/or other satDNA sequences, or (ii) sequences that are, together with satDNA repeats, assigned as parts of TEs of the *Helitron* superfamily. Both patterns suggest tight interconnection between satDNAs and TEs. In addition, detection of individual satDNA monomers in some genomic fragments is indicative of hypothetical transposition-related ability of satDNA sequences to be relocated independently throughout the genome. Comparably, fragmented satDNA monomers alternating with anonymous sequences in some array flanking regions can be a consequence of cut-and-replace events involved in rapid deterioration of satDNA monomers at array ends.

Altogether, our results indicate a close link between satDNAs and TEs in examined mollusc species, highlighting integration of the two sequence types into a complex network able to shape genomic repetitive environment and alter the entire genome. Accumulating knowledge about mobility and interspersion patterns of satDNA repats will also shift the focus of the future work, from bulk analyses of satDNA monomers recovered from long arrays towards studies targeted on satDNA repeats interspersed in the genome and closely associated with diverse genomic sequences.

## Additional files

Additional file 1: Table S1. List of all analyzed genomic fragments, their composition and related sequences. (DOCX 45 kb)
Additional file 2: Table S2. Positions of analyzed Crassostrea gigas genomic fragments in scaffolds. (DOCX 14 kb)
Additional file 3: Figure S1. Phylogenetic analysis of satDNA monomers. (PDF 112 kb)
Additional file 4: Figure S2. SatDNA junctions. (PDF 22 kb)
Additional file 5: Table S3. List of previously described satDNAs detected in this work. (DOCX 16 kb)
