# Supplementary material for: Adjacent sequences disclose potential for intra-genomic dispersal of satellite DNA repeats and suggest a complex network with transposable elements
Source: BMC Genomics. 2016 Dec 6;17:997. doi: 10.1186/s12864-016-3347-1 (PMC5139131; doi:10.1186/s12864-016-3347-1)
Supplement: Additional file 1: Table S1. — List of all analyzed genomic fragments, their composition and related sequences. (DOCX 45 kb) [file 12864_2016_3347_MOESM1_ESM.docx]

**Additional file 1: Table S1**

List of all analyzed genomic fragments, their composition and related sequences

1. *Ruditapes philippinarum*

| **Fragment** *(acc. number)* | **Satellite DNA** | **Transposable element** | | | **Other observed sequence similarities** | **Fragment lenght** |
| --- | --- | --- | --- | --- | --- | --- |
|  |  | **DNA transposon** | **Retrotransposon** | |  |  |
|  |  |  | **LTR** | **Non-LTR** |  |  |
| P1*  *(KU682300)* | 338-663 ***phBgl*II400** | 168 -282 **Helitron-N2_SP** |  |  |  | 663 |
| P2*  *(KU682305)* | 311-718 ***phBgl*II400**  13-55 **DTRS sat** |  | 525-602  **Copia-104_GM-I** |  |  | 718 |
| P3*  *(KU682306)* | 1-223 ***phBgl*II400** | 500 -691 **Ginger1-11_HM** 1267-1387 [**npiggyBac-3_Hmel**](http://www.girinst.org/protected/repbase_extract.php?access=npiggyBac-3_Hmel&format=EMBL) 1569-1638 [**Mariner-60_Hsal**](http://www.girinst.org/protected/repbase_extract.php?access=Mariner-60_HSal&format=EMBL) | 131-180 [**Gypsy-22_FV-I**](http://www.girinst.org/protected/repbase_extract.php?access=Gypsy-22_FV-I&format=EMBL) |  |  | 1642 |
| P4  *(KU682307)* | 1-403 ***phBgl*II400** |  |  |  |  | 403 |
| P5 | 1-97 ***phBgl*II400** |  |  |  |  | 97 |
| P12*  *(KU682301)* | 1-316 ***phBgl*II400** |  |  |  |  | 656 |
| P16*  *(KU682303)* | 143-241***phBgl*II400** | 1-83 **Helitron-N1_SP** |  |  |  | 241 |
| P17 | 1-97 ***phBgl*II400** |  |  |  |  | 97 |
| P18*  *(KU682304)* | 770-1083 ***phBgl*II400**  1890-1985 ***phBgl*II400**  2008-3433 **BIV sat** | 1258-1301 [**Academ-1_Dpulex**](http://www.girinst.org/protected/repbase_extract.php?access=Academ-1_Dpulex&format=EMBL) 2232-2351 [**Helitron-N2e_CGi**](http://www.girinst.org/protected/repbase_extract.php?access=Helitron-N2e_CGi&format=EMBL) 2356-2467 [**Harbinger-7_CGi**](http://www.girinst.org/protected/repbase_extract.php?access=Harbinger-7_CGi&format=EMBL) 2569-2875 [**Helitron-N2f_CGi**](http://www.girinst.org/protected/repbase_extract.php?access=Helitron-N2f_CGi&format=EMBL) 2920-3017 [**Helitron-N2e_CGi**](http://www.girinst.org/protected/repbase_extract.php?access=Helitron-N2e_CGi&format=EMBL) 3610-3688 **EnSpm-5_VV** | 523-580 [**Copia-2_Ano-I**](http://www.girinst.org/protected/repbase_extract.php?access=Copia-2_Ano-I&format=EMBL) | 130-204 **L1-56_XT** 1088-1136 [**L2-5_LCh**](http://www.girinst.org/protected/repbase_extract.php?access=L2-5_LCh&format=EMBL) | 514-891,1116-1583 RNA-directed DNA polymerase from mobile element jockey 1083-1577  Pol protein | 3995 |
| P34* | 32-128 ***phBgl*II400** |  |  |  |  | 128 |
| P35* | 2-105 ***phBgl*II400** |  |  |  |  | 145 |
| P42*  *(KU682308)* | 153-251 ***phBgl*II400** |  |  |  |  | 251 |
| P43*  *(KU682309)* | 332-428 ***phBgl*II400** |  |  |  |  | 428 |
| P46*  *(KU682310)* | 1-156 **DTHS3**  1906-2004 ***phBgl*II400** | 147-256 **CVA**  1008-1168 **Helitron-1_DPe** |  | 1217-1266 **Crack-1_NV** | 15-177 *Spisula solidissima* gene for Zn finger protein Sso-Zic | 2004 |
| P47 | 1-143 ***phBgl*II400** |  |  |  |  | 143 |
| P49  *(KU682311)* | 1-307 ***phBgl*II400** |  |  |  |  | 307 |
| PD53F*  *(KU682312)* | 1187-1666 **DTHS3** | 1582-1628 **Helitron-N9_CGi** |  |  |  | 1666 |
| PD53R*  *(KU682313)* | 1-682 **DTHS3** |  |  | 109-152 [**SART-6_APi**](http://www.girinst.org/protected/repbase_extract.php?access=SART-6_APi&format=EMBL) |  | 911 |
| PD60* | 1-106 ***phBgl*II400** |  |  |  |  | 160 |

1. *Ruditapes decussatus*

| **Fragment** *(acc. number)* | **Satellite DNA** | **Transposable element** | | | **Other observed sequence similarities** | **Fragment lenght** |
| --- | --- | --- | --- | --- | --- | --- |
|  |  | **DNA transposon** | **Retrotransposon** | |  |  |
|  |  |  | **LTR** | **Non-LTR** |  |  |
| D7F*  *(KU682298)* | 559-646 **DTHS3** |  |  |  |  | 918 |
| D12*  *(KU682294)* | 149-221, 485-552 **DTHS3** |  |  | \| 222-484 **RUDI** \|  \| \| --- \| --- \| |  | 1003 |
| D17  *(KU682295)* | 1-837 **BIV160** |  |  |  |  | 837 |
| D19  *(KU682296)* | 1-660 **BIV160** |  |  |  |  | 660 |
| D26*  *(KU682297)* | 351-545 **DTHS3** | 223-298 [**hAT-18_Crp**](http://www.girinst.org/protected/repbase_extract.php?access=hAT-18_Crp&format=EMBL) 341-384 [**LIMPET1**](http://www.girinst.org/protected/repbase_extract.php?access=LIMPET1&format=EMBL) |  |  |  | 545 |
| D32 | 1 -145 **DTHS3** |  |  |  |  | 145 |
| DP36*  *(KU682299)* | 1-149 **DTHS3** |  |  |  | 27-148 *S. solidissima* cnRNA86 | 224 |

1. *Donax trunculus*

| **Fragment** *(acc. number)* | **Satellite DNA** | **Transposable element** | | | **Other observed sequence similarities** | **Fragment lenght** |
| --- | --- | --- | --- | --- | --- | --- |
|  |  | **DNA transposon** | **Retrotransposon** | |  |  |
|  |  |  | **LTR** | **Non-LTR** |  |  |
| DTC4Alu  *(KU682290)* | 1-397 **Dt-BIV160** |  |  |  |  | 397 |
| DTC17AluF* *(KC981731)* | 1-55 **Dt-BIV160** | 132-209 [**ISL2EU-13_CGi**](http://www.girinst.org/protected/repbase_extract.php?access=ISL2EU-13_CGi&format=EMBL) |  | 450-504 **CR1-5_NV** |  | 870 |
| DTC32Alu*  *(KU682285)* | 874-1453 **DTHS3** |  |  | 244-327 [**Crack-1_LG**](http://www.girinst.org/protected/repbase_extract.php?access=Crack-1_LG&format=EMBL) 370-487  [**Daphne-21_HMa**](http://www.girinst.org/protected/repbase_extract.php?access=Daphne-21_HMa&format=EMBL) | 230-601 reverse transcriptase from mobile element  jockey (Drosophila) | 1456 |
| DTC34Alu*  *(KU682286)* | 1-57 **DTHS1** | 783-828 **MIRAGE1** 161-421 **DTC M1** 708-867 **DTC M2** |  | 19-56 **Coprina_Cc1** |  | 1406 |
| DTC38Alu*  *(KU682287)* | 213-283 **DTE** | 3-103 **DTC M2** |  | 183-221 **Vingi-1_Tcas** |  | 283 |
| DTC50Alu  *(KU682291)* | 1-328 **Dt-BIV160** |  |  |  |  | 328 |
| DTC51Alu*  *(KU682292)* | 139-306 **Dt-BIV160** | 185-305 [**Helitron-N2d_CGi**](http://www.girinst.org/protected/repbase_extract.php?access=Helitron-N2d_CGi&format=EMBL) |  |  |  | 306 |
| DTC52Alu *(KC981735)* | 1-720 **Dt-BIV160** |  |  |  |  | 720 |
| 84-19F*  *(KU682284)* | 1-147 **DTHS3** | 189-377 **DTC M2** |  |  |  | 626 |
| 84-35*  *(KC981682)* | 34-203 **Dt-BIV160** | 17-196 [**Helitron-N2d_CGi**](http://www.girinst.org/protected/repbase_extract.php?access=Helitron-N2d_CGi&format=EMBL)  198-237 [**Helitron-2_PSt**](http://www.girinst.org/protected/repbase_extract.php?access=Helitron-2_PSt&format=EMBL)  410-694 **DTC84**  569-646 [**Helitron-N26_CGi**](http://www.girinst.org/protected/repbase_extract.php?access=Helitron-N26_CGi&format=EMBL)  856-902 **DTC M1** |  |  |  | 1432 |

1. *Crassostrea gigas*

| **Fragment** | **Satellite DNA** | **Transposable element** | | | **Other observed sequence similarities** | **Fragment lenght** |
| --- | --- | --- | --- | --- | --- | --- |
|  |  | **DNA transposon** | **Retrotransposon** | |  |  |
|  |  |  | **LTR** | **Non-LTR** |  |  |
| R_Cg 1* | 501 - 867 **Cg170** | 501-1096 **Helitron-N55_CGi**  1218-1319 **Helitron-N21_CGi**  1-75 **DNA3-10_CGi** |  | 281-500 **CR1-10_CGi** |  | 1322 |
| R_Cg 2* | 498 - 1048 **Cg170** | 69-491 **Helitron-N55_CGi**  493-1266 **Helitron-N55_CGi** 1267-1448 **Helitron-N40_CGi** 1-68 **Helitron-N28_CGi** |  |  |  | 1448 |
| R_Cg 3* | 475 - 948 **Cg170** | 475-1151 **Helitron-N55_CGi** 127-460 **Helitron-N3_CGi**  3-126 **Helitron-N1B_CGi**  1219-1427 **DNA9-4_CGi** |  |  |  | 1448 |
| R_Cg 4* | 501 - 3206 **Cg170** | 3305-3564 **Helitron-N22_CGi** 562-1394 **Helitron-N2_CGi** 1,411-3,3 **Helitron-N2_CGi** 492-561 **Helitron-N1B_CGi** 171-259 **EnSpm-5_HM** |  |  |  | 3564 |
| R_Cg 5 rev* | 1524 - 505 **Cg170** | 1544-1698 **Mariner-21_CGi**  1-258 **Helitron-N18_CGi**  259-1536 **Helitron-N2_CGi**  1,813-1915 **DNA4-30_CGi** |  |  |  | 2013 |
| R_Cg 6* | 488 - 2012 **Cg170** | 244-370 **Mariner-1_RC**  2013-2134 **Helitron-N2f_CGi** 488-1996 **Helitron-N1B_CGi** |  |  |  | 2502 |
| R_Cg 7* | 498 - 1060 **Cg170** | 1061-155 **ISL2EU-4N1_CGi** 261-495 **Helitron-N7B_CGi** 496-106 **Helitron-N2_CGi** |  |  |  | 1562 |
| R_Cg 8* | 501 - 1808 **Cg170** | 16-495 **Helitron-N2e_CGi**  134-479 **Helitron-N2_CGi**  11-68 **Helitron-N1B_CGi**  498-1808 **Helitron-N1B_CGi** 1809-2306 **Helitron-7_CGi** |  |  |  | 2308 |
| R_Cg 9 rev* | 916 - 420 **Cg170** | 1-139 **Helitron-N51_CGi**  140-974 **Helitron-N2_CGi** 1057-1312 **DNA2-34_CGi** |  |  |  | 1395 |
| R_Cg 10 rev* | 380 - 6 **Cg170** | 1-170 **Kolobok-N2_CGi**  171-881 **Helitron-N2_CGi** |  |  |  | 881 |
| T_Cg1 rev* | 501 - 1541 **Cg170** | 59-155 **DNA2-5_CGi**  247-2040 **Helitron-N55_CGi** |  |  |  | 2041 |
| T_Cg2 rev* | 501 - 1373 **Cg170** | 11-62 **Helitron-N17B_CGi**  118-260 **ISL2EU-N3_CGi**  262-1873 **Helitron-N55_CGi** |  |  |  | 1873 |
| T_Cg3 rev* | 1 - 1271 **Cg170** | 1-1475 **Helitron-N2_CGi** |  |  |  | 1771 |
| T_Cg4* | 501 - 1544 **Cg170** | 1-237 **Helitron-N7B_CGi**  238-1666 **Helitron-N55_CGi**  1677-1783 **Helitron-4_CGi**  1802-1880 **DNA-10_CGi**  1884-2029 **Helitron-N52_CGi** |  |  |  | 2044 |
| T_Cg5* | 501 - 2867 **Cg170** | 3-253 **Helitron-N29_CGi**  254-1306 **Helitron-N2_CGi**  1307-3204 **Helitron-N1B_CGi**  3207-3326 **Mariner-N6_CGi**  3327-3367 **Zator-3_CGi** |  |  |  | 3367 |
| T_Cg6* | 501 - 1367 **Cg170** | 250-1866 **Helitron-N55_CGi** |  |  |  | 1867 |
| T_Cg7* | 501 - 2375 **Cg170** | 2-92 **Mariner-3_CGi**  162-219 **ISL2EU-6_HM**  250-2716 **Helitron-N2_CGi**  2719-2874 **Crypton-N13_CGi** |  |  |  | 2875 |
| T_Cg8* | 521 - 2228 **Cg170** | 1-266 **Helitron-N2d_CGi**  274-2388 **Helitron-N2_CGi**  2389-2728 **Helitron-N55_CGi** |  |  |  | 2728 |
| T_Cg9* | 1 - 468 **Cg170** | 1-968 **Helitron-N55_CGi** |  |  |  | 968 |
| T_Cg10* | 501 - 1374 **Cg170** | 55-145 **Academ-1_Hrobusta**  283-1531 **Helitron-N55_CGi**  1532-1729 **Helitron-4_CGi** |  |  |  | 1874 |
