# Supplementary material for: Adjacent sequences disclose potential for intra-genomic dispersal of satellite DNA repeats and suggest a complex network with transposable elements
Source: BMC Genomics. 2016 Dec 6;17:997. doi: 10.1186/s12864-016-3347-1 (PMC5139131; doi:10.1186/s12864-016-3347-1)
Supplement: Additional file 2: Table S2. — Positions of analyzed Crassostrea gigas genomic fragments in scaffolds. (DOCX 14 kb) [file 12864_2016_3347_MOESM2_ESM.docx]

**Additional file 2: Table S2**

Positions of analyzed *Crassostrea gigas* genomic fragments in scaffolds

| Fragment | Scaffold | Positions (bp) | Sequence length (bp) |
| --- | --- | --- | --- |
| T_Cg1 rev | scaffold1252 | 154941.-152901. | 2041 |
| T_Cg2 rev | scaffold301 | 890196.-888324. | 1873 |
| T_Cg3 rev | scaffold36092 | 30216.-28446. | 1771 |
| T_Cg4 | scaffold363 | 137443.-139486. | 2044 |
| T_Cg5 | scaffold402 | 275414.-278780. | 3367 |
| T_Cg6 | scaffold41136 | 25433.-27299. | 1867 |
| T_Cg7 | scaffold42566 | 8363.-11237. | 2875 |
| T_Cg8 | scaffold43364 | 22548.-25275. | 2728 |
| T_Cg9 | scaffold750 | 245442.-246409. | 968 |
| T_Cg10 | scaffold86 | 1686140.-1688013. | 1874 |
| R_Cg1 | C31762 | 2326.-3647. | 1322 |
| R_Cg2 | C34372 | 11621.-13068. | 1448 |
| R_Cg3 | scaffold156 | 1149903.-1151350. | 1448 |
| R_Cg4 | scaffold191 | 54763.-58326. | 3564 |
| R_Cg5 rev | scaffold339 | 151937.-149925. | 2013 |
| R_Cg6 | scaffold1307 | 631195.-633696. | 2502 |
| R_Cg7 | scaffold1511 | 16854.-18415. | 1562 |
| R_Cg8 | scaffold35900 | 11468.-13775. | 2308 |
| R_Cg9 rev | scaffold1703 | 104095.-102701. | 1395 |
| R_Cg10 rev | C24428 | 881.-1. | 881 |
