# Supplementary material for: Adjacent sequences disclose potential for intra-genomic dispersal of satellite DNA repeats and suggest a complex network with transposable elements
Source: BMC Genomics. 2016 Dec 6;17:997. doi: 10.1186/s12864-016-3347-1 (PMC5139131; doi:10.1186/s12864-016-3347-1)
Supplement: Additional file 3: Figure S1. — Phylogenetic analysis of satDNA monomers. (PDF 112 kb) [file 12864_2016_3347_MOESM3_ESM.pdf]

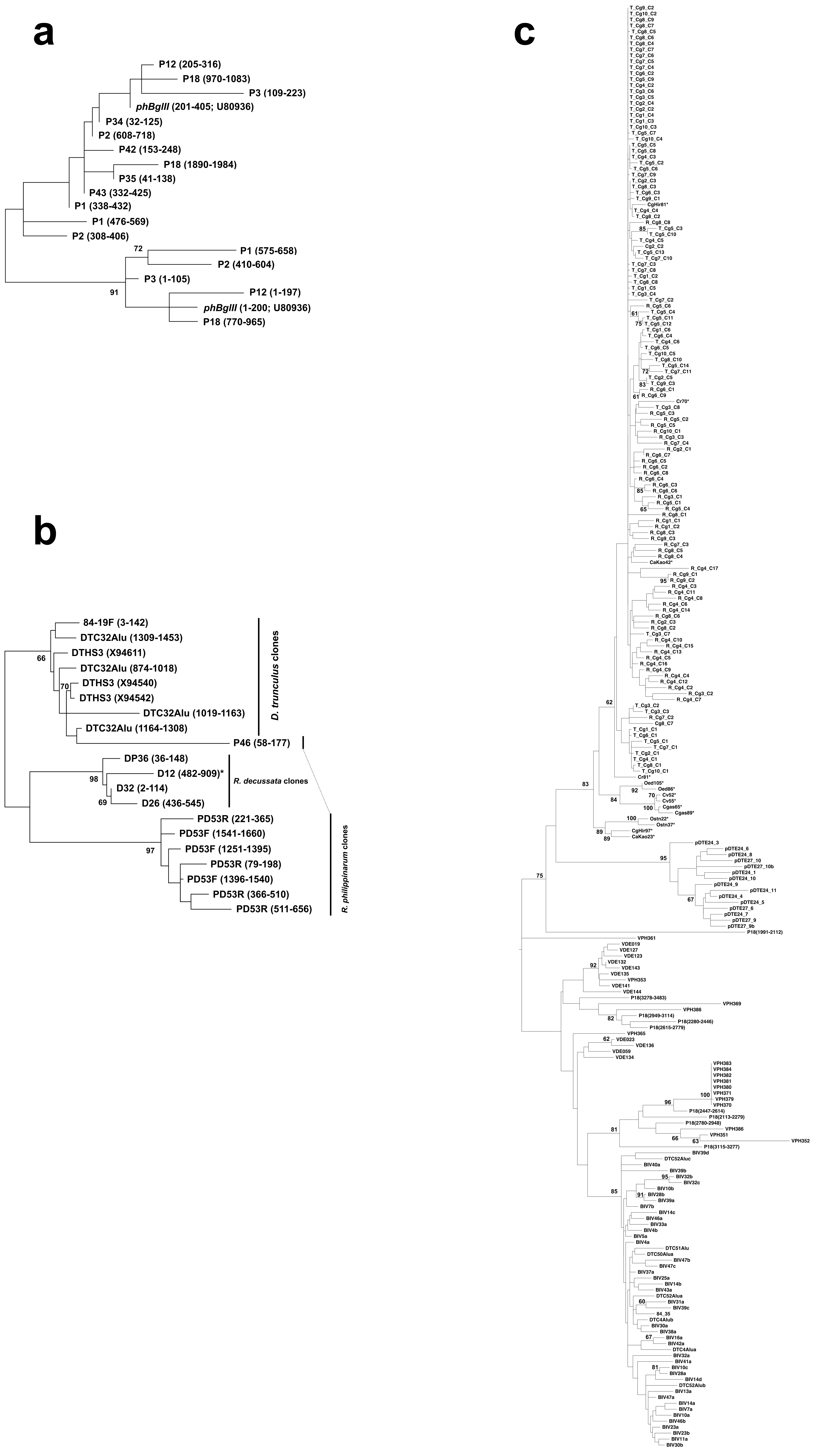
a
P12 (205-316)
P18 (970-1083)
P3 (109-223)
phBglII (201-405; U80936)
P34 (32-125)
P2 (608-718)
P42 (153-248)
P18 (1890-1984)
P35 (41-138)
P43 (332-425)
P1 (338-432)
P1 (476-569)
P2 (308-406)
72
P1 (575-658)
P2 (410-604)
P3 (1-105)
91
P12 (1-197)
phBglII (1-200; U80936)
P18 (770-965)
b
84-19F (3-142)
DTC32Alu (1309-1453)
66
DTHS3 (X94611)
DTC32Alu (874-1018)
70
DTHS3 (X94540)
DTHS3 (X94542)
DTC32Alu (1019-1163)
DTC32Alu (1164-1308)
D. trunculus clones
P46 (58-177)
DP36 (36-148)
98
D12 (482-909)*
D32 (2-114)
69
D26 (436-545)
R. decussata clones
PD53R (221-365)
PD53F (1541-1660)
97
PD53F (1251-1395)
PD53R (79-198)
PD53F (1396-1540)
PD53R (366-510)
PD53R (511-656)
R. philippinarum clones
c
T_Cg9_C2
T_Cg10_C2
T_Cg8_C9
T_Cg8_C7
T_Cg8_C5
T_Cg8_C6
T_Cg8_C4
T_Cg7_C7
T_Cg7_C6
T_Cg7_C5
T_Cg7_C4
T_Cg6_C2
T_Cg5_C9
T_Cg4_C2
T_Cg3_C6
T_Cg3_C5
T_Cg2_C4
T_Cg2_C2
T_Cg1_C4
T_Cg1_C3
T_Cg10_C3
T_Cg5_C7
T_Cg10_C4
T_Cg5_C5
T_Cg5_C8
T_Cg4_C3
T_Cg5_C2
T_Cg5_C6
T_Cg7_C9
T_Cg2_C3
T_Cg8_C3
T_Cg6_C3
T_Cg9_C1
CgHir81*
T_Cg4_C4
T_Cg8_C2
R_Cg8_C8
85
T_Cg5_C3
T_Cg5_C10
T_Cg4_C5
Cg2_C2
T_Cg5_C13
T_Cg7_C10
T_Cg7_C3
T_Cg7_C8
T_Cg1_C2
T_Cg8_C8
T_Cg1_C5
T_Cg3_C4
T_Cg7_C2
R_Cg5_C6
T_Cg5_C4
61
T_Cg5_C11
75
T_Cg5_C12
T_Cg1_C6
T_Cg6_C4
T_Cg4_C6
T_Cg6_C5
T_Cg10_C5
T_Cg8_C10
T_Cg5_C14
72
T_Cg7_C11
T_Cg2_C5
83
T_Cg9_C3
R_Cg6_C1
61
R_Cg6_C9
Cr70*
T_Cg3_C8
R_Cg5_C3
R_Cg5_C2
R_Cg5_C5
R_Cg10_C1
R_Cg3_C3
R_Cg7_C4
R_Cg2_C1
R_Cg6_C7
R_Cg6_C5
R_Cg6_C2
R_Cg6_C8
R_Cg6_C4
R_Cg6_C3
85
R_Cg6_C6
R_Cg3_C1
R_Cg5_C1
65
R_Cg5_C4
R_Cg8_C1
R_Cg1_C1
R_Cg1_C2
R_Cg8_C3
R_Cg9_C3
R_Cg7_C3
R_Cg8_C5
R_Cg8_C4
CaKao42*
R_Cg4_C17
R_Cg9_C1
95
R_Cg9_C2
R_Cg4_C3
R_Cg4_C11
R_Cg4_C8
R_Cg4_C6
R_Cg4_C14
R_Cg8_C6
R_Cg2_C3
R_Cg8_C2
T_Cg3_C7
R_Cg4_C10
R_Cg4_C15
R_Cg4_C13
R_Cg4_C5
R_Cg4_C16
R_Cg4_C9
R_Cg4_C4
R_Cg4_C12
R_Cg4_C2
R_Cg3_C2
R_Cg4_C7
62
T_Cg3_C2
T_Cg3_C3
R_Cg7_C2
Cg8_C7
T_Cg1_C1
T_Cg6_C1
T_Cg5_C1
T_Cg7_C1
T_Cg2_C1
T_Cg4_C1
T_Cg8_C1
T_Cg10_C1
Cr81*
83
Oed105*
92
Oed86*
84
70
Cv52*
Cv55*
100
Cgas65*
Cgas89*
100
Ostn22*
Ostn37*
89
CgHir97*
89
CaKao23*
pDTE24_3
pDTE24_6
pDTE24_8
pDTE27_10
95
pDTE27_10b
pDTE24_1
pDTE24_10
75
pDTE24_9
pDTE24_11
67
pDTE24_4
pDTE24_5
pDTE27_6
pDTE24_7
pDTE27_9
pDTE27_9b
P18(1991-2112)
VPH361
VDE019
VDE127
VDE123
VDE132
92
VDE143
VDE135
VPH353
VDE141
VDE144
P18(3278-3483)
VPH369
VPH386
82
P18(2949-3114)
P18(2280-2446)
P18(2615-2779)
VPH365
62
VDE023
VDE136
VDE059
VDE134
VPH383
VPH384
VPH382
VPH381
VPH380
100
VPH371
VPH379
96
VPH370
P18(2447-2614)
P18(2113-2279)
81
P18(2780-2948)
VPH386
VPH351
66
63
VPH352
P18(3115-3277)
BIV39d
DTC52Aluc
BIV40a
BIV39b
95
BIV32b
BIV32c
85
BIV10b
91
BIV28b
BIV39a
BIV7b
BIV14c
BIV46a
BIV33a
BIV4b
BIV5a
BIV4a
DTC51Alu
DTC50Alua
BIV47b
BIV47c
BIV37a
BIV25a
BIV14b
BIV43a
DTC52Alua
60
BIV31a
BIV39c
84_35
DTC4Alub
BIV30a
BIV38a
67
BIV16a
BIV42a
DTC4Alua
BIV32a
BIV41a
81
BIV10c
BIV28a
BIV14d
DTC52Alub
BIV13a
BIV47a
BIV14a
BIV7a
BIV10a
BIV46b
BIV23a
BIV23b
BIV11a
BIV30b
