# Supplementary figures and images for: Adjacent sequences disclose potential for intra-genomic dispersal of satellite DNA repeats and suggest a complex network with transposable elements
Source: BMC Genomics. 2016 Dec 6;17:997. doi: 10.1186/s12864-016-3347-1 (PMC5139131; doi:10.1186/s12864-016-3347-1)

a

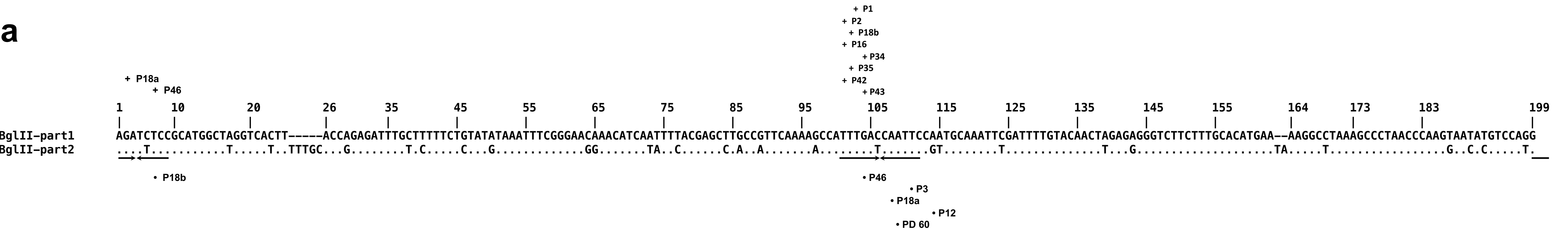

b

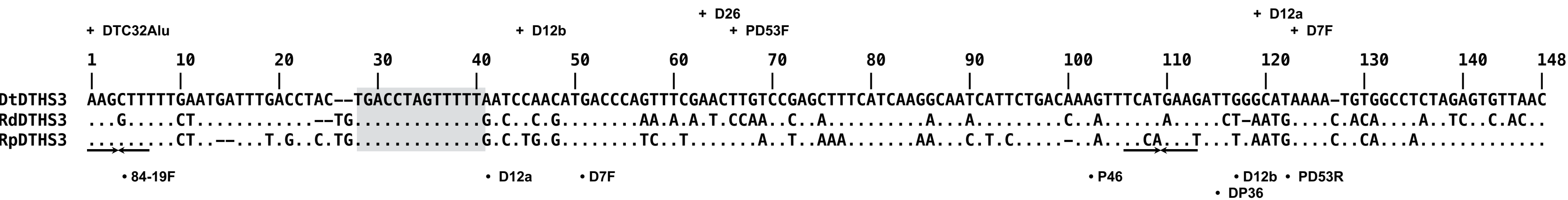

c

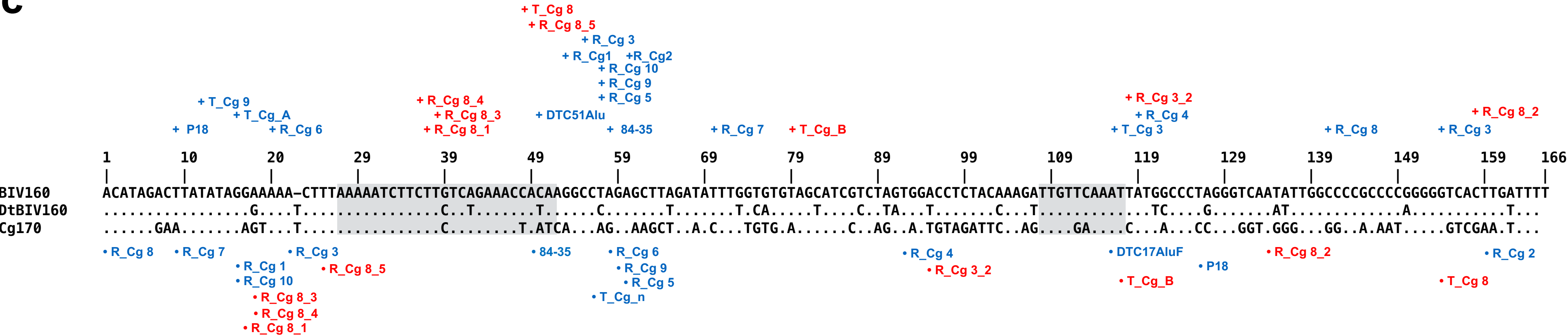

Supplement: Additional file 4: Figure S2. — SatDNA junctions. (PDF 22 kb) [file 12864_2016_3347_MOESM4_ESM.pdf]
