# Supplementary material for: Adjacent sequences disclose potential for intra-genomic dispersal of satellite DNA repeats and suggest a complex network with transposable elements
Source: BMC Genomics. 2016 Dec 6;17:997. doi: 10.1186/s12864-016-3347-1 (PMC5139131; doi:10.1186/s12864-016-3347-1)
Supplement: Additional file 5: Table S3. — List of previously described satDNAs detected in this work. (DOCX 16 kb) [file 12864_2016_3347_MOESM5_ESM.docx]

| Species SatDNA Monomer length / bp Genome content/% Reference |
| --- |
|  |
| *Donax trunculus* DTHS1 162 0.043 [43]  *Donax trunculus* DTHS3 145 0.035 [43]  *Donax trunculus* DTE 155 0.32 [37]  *Donax trunculus* DTRS 288 < 0.1 [44]  *Ruditapes decussatus* BIV160 158-165 2 [34]  *Ruditapes philippinarum* BIV160 158-165 < 0.03 [34] (and other bivalves)  *Ruditapes philippinarum* phBglII400 405 ND [42]  *Crassostrea gigas* Cg170 (*Hin*dIII) 166 1-4 [45-47] |

**Additional file 5: Table S3**

List of previously described satDNAs detected in this work

ND, not determined
